# A nomogram predicting the risk of postoperative pneumonia after esophagectomy in esophageal carcinoma

**DOI:** 10.3389/fmed.2025.1553163

**Published:** 2025-06-11

**Authors:** Binlie Chen, Weiqi Ke, Meizhen Li

**Affiliations:** ^1^Department of Gastrointestinal Surgery, The First Affiliated Hospital of Shantou University Medical College, Shantou, Guangdong, China; ^2^Department of Anesthesiology, The First Affiliated Hospital of Shantou University Medical College, Shantou, Guangdong, China

**Keywords:** nomogram, predict, risk, pneumonia, esophagectomy

## Abstract

**Background:**

Pneumonia is a common complication following esophagectomy, which is related with an increased risk of mortality and hospitalization. This condition not only prolongs hospital stays but also raises healthcare costs. The aim of this study was to identify risk variables and develop a nomogram for predicting postoperative pneumonia (PP).

**Methods:**

A total of 647 individuals who had esophageal cancer surgery between January 1, 2010, and December 31, 2020, were involved in this study. We used least absolute shrinkage and selection operator (LASSO) regression for screening the optimal predictive factors and subsequently developed a nomogram using the selected factors. Verification through the use of 500 bootstrap resampling techniques. To assess the nomogram’s discriminating power, we used the calibration plot, receiver operating characteristic (ROC) curve, and decision curve analysis (DCA).

**Results:**

According to the standard error criteria of non-zero coefficients of LASSO and multivariate logistic regression analyses, age, smoking, double-lumen endotracheal tube (DLET), combined intravenous and inhalation anesthesia (CIIA), and vasoactive drugs usage are independent risk indicators of PP. Based on these five predictors we created a nomogram. The area under the of nomogram for the ROC curve was 0.665 (95% CI: 0.620–0.704) in development and 0.691 (95%CI: 0.654–0.726) in 500 bootstraps resample validation. Additionally, the calibration curves showed a high degree of agreement between the actual and predicted probabilities. DCA displayed that the predictive model had a net benefit when the risk thresholds were 0.17–0.61.

**Conclusion:**

This study developed an intuitive nomogram model to predict postoperative pneumonia in esophageal cancer patients based on age, smoking history, DLET, CIIA, and vasoactive medication usage. Proper anesthesia, ETT type, smoking cessation, and timely vasoactive medication use can lower risks. Further external validation and large-scale studies are needed.

## Background

Esophageal carcinoma (EC) is the sixth largest cause of cancer mortality worldwide (1). Esophagectomy is still the most successful therapeutic method, however preoperative chemotherapy or chemoradiotherapy may be a viable choice in the treatment of esophageal cancer (2). Up to 40% of patients with esophageal cancer get postoperative pneumonia (PP) following esophagectomy (3, 4), which lengthens hospital stays, raises costs, and increases mortality risk (5). Meanwhile, in salvage esophagectomy, PP was the vital parameter to predict the overall survival (6). Therefore, it is essential to improve prevention and treatment of PP during the perioperative management of esophagectomy in esophageal carcinoma with a high mortality.

There are several factors that may contribute to pneumonia after esophagectomy, including advanced age, smoking history, pulmonary diseases, malnutrition, neoadjuvant chemotherapy, long operation time and one lung ventilation with double lumen endotracheal tube etc (2, 7, 8). However, factors that may promote pneumonia are still controversial. Finding potential high-risk factors, avoiding PP, and implementing best care techniques are essential to improving the prognosis of patients with EC after esophagectomy. In clinical practice, a clinical predictive model for PP after esophagectomy is urgently required. Nomogram as one of available models, can provide a precise, personalized evidence-based risk evaluation. To the best of our knowledge, however, only few clinical prediction models of nomogram have been applied to predict the likelihood of PP in patients who had radical esophagectomy. In previous research on prediction models, the sample size was relatively small (9, 10). Moreover, the included factors were relatively few. One of them (10) only contained two predictors and did not cover the entire perioperative surgical anesthesia factors and laboratory test results. Therefore, a more comprehensive prediction model for postoperative pneumonia after esophageal cancer is urgently needed in this research field.

In order to create and validate a nomogram of PP in patients with EC undergoing esophageal excision based on independent risk factors, we set out to investigate preoperative patients, anesthetic, and surgical risk factors for PP.

## Methods

### Study patients

We retrospectively analyzed the patients who had radical esophagectomy of the First Affiliated Hospital of Shantou University Medical College in Guangdong, China, between January 1, 2010 and December 31, 2020. Patients with missing medical records, those who were discharged against medical advice or died after surgery, those who had an unexpected second surgery, those who canceled a surgery or combined operation with other locations, and those who had a postoperative pathological examination that turned up non-esophageal cancer were excluded.

In accordance with the Declaration of Helsinki and the relevant TRIPOD guidelines, this study was approved by the Ethics Committee of the First Affiliated Hospital of Shantou University Medical College (NO. B-2021-249). Anonymous data analysis was conducted, and informed consent was not required.

### Surgical and anesthetic techniques options

Esophageal resection techniques can be categorized into two main approaches: traditional open esophagectomy (OE) and minimally invasive esophagectomy (MIE). MIE can further be divided based on the anastomosis location. The McKeown MIE technique involves thoracoscopic esophageal resection, laparoscopic gastric mobilization, and cervical anastomosis, whereas the Ivor-Lewis MIE technique includes a thoracic phase for esophageal resection and intrathoracic esophagogastric anastomosis. Typically, tumors in the upper and middle thoracic regions are better suited for McKeown MIE, while those in the lower thoracic region are more appropriate for Ivor-Lewis MIE or open esophagectomy. In our hospital, the most commonly performed procedures are open esophagectomy (either left or right transthoracic) and McKeown MIE (via the right transthoracic route). The Ivor-Lewis MIE is less frequently performed due to the challenges associated with lymph node dissection around the left recurrent laryngeal nerve and managing potential anastomotic leaks. Overall, McKeown MIE is the preferred method in most cases. However, when the tumor is near the gastric cardia, open esophagectomy is chosen. For anesthesia during esophageal resection, either general anesthesia (GA) or a combination of GA and thoracic epidural anesthesia (E-GA) may be used. After standard induction of general anesthesia, a double-lumen endotracheal tube (DLET) is inserted for left lung collapse in OE, while single-lumen endotracheal intubation is used for two-lung ventilation in MIE. If a patient planned for DLET placement has a difficult airway, bronchial occluder can be considered for one-lung ventilation following awake intubation using fiber optic bronchoscopy through a single-lumen endotracheal tube (SLET). The choice of anesthesia maintenance drugs by anesthesiologists is mainly divided into total intravenous anesthesia (TIVA), combined intravenous and inhalation anesthesia (CIIA). Intraoperative hypotension is mainly maintained by intravenous infusion of norepinephrine and norepinephrine. The anesthesia plan is tailored by the anesthesiologist in collaboration with the surgeon and patient, based on a comprehensive preoperative assessment. Perioperative management is individualized for each patient by the anesthesiologist.

### Definition of postoperative pneumonia

During the first 2 weeks following esophagectomy, clinical signs and imaging are used to diagnosis postoperative pneumonia: (1) with purulent discharges, fever, chest tightness, productive cough, leukocyte count > 10.0 × 10^9^ /L or < 4.0 × 10^9^/L; (2) with postoperative imaging showing new or progressive development, consolidation, cavitation, or persistent pulmonary infiltrate shadows (11).

### Sample size calculation

Based on the events per variable (EPV) principle (12, 13), the number of variables is 21 and the EPV is set at 10. The sample size calculation formula is as follows (14). The incidence of pneumonia in this study is 32.77%. Substituting into the formula, the required sample size is 312. The sample size of this study is 647, which is sufficient.


$$\text{Sample size}=\frac{\text{Number of variables}\times \text{EPV}}{1-\text{Incidence Rate}}$$

### Data collection

As explained below, we added the following variables, which are factors related to perioperative risk that impact PP: (1) Factors associated with preoperative patients: baseline demographics (age, gender, smoking and alcohol use), comorbid conditions (diabetes, hypertension, or pulmonary disease), neoadjuvant chemotherapy, tumor location, and laboratory test results [albumin (ALB), hemoglobin (Hb)]; (2) Anesthesiologist-related variables: surgical technique (OE, MIE), and operating time (OT); (3) Anesthesia-related variables: physical status of the American Society of Anesthesiologists (ASA), type of anesthesia (GA, E-GA), continuous anesthesia (TIVA, CIIA), type of endotracheal tube (ETT) [(DLET, SLET) (15), vasoactive drug usage, perioperative fluid volume (PFV), estimated blood loss (EBL), and patient-controlled analgesia (PCA)] [patient controlled intravenous analgesia (PCIA), patient controlled epidural analgesia (PCEA)].

### Model establishment and validation

Potential risk variables were roughly clarified using univariate analysis. A collinearity analysis was performed on each independent variable, and the variable with a variance inflation factor (VIF) more than 10 was eliminated (16). To identify the optimal predictive factors for PP, we employed least absolute shrinkage and selection operator (LASSO) regression for selection and subsequently developed a nomogram using the selected factors. The model was estimated using three metrics: (1) The discriminatory capacity of the model was assessed using receiver operating characteristic (ROC) curve analysis. The accuracy of our model was further confirmed by bootstrap validation using computer resampling for 500 repetitions of simple random sampling with replacement; (2) The calibration curve detected the concentricity between the model probability curve and ideal curve; and (3) The decision curve analysis (DCA) of net benefit curve was used to evaluate the clinical usefulness of our model.

### Statistical analysis

In contrast to categorical variables, which are presented as numbers and percentages, continuous data are presented as the mean ± SD or median (min-max value). The t-test was used to test data with a normal distribution, and the non-parametric test of two independent samples was used to test data with an abnormal distribution. The χ2 test was used to assess categorical variables. We employed LASSO regression for screening the optimal predictive factors and subsequently developed a nomogram using the selected factors. The nomogram’s effectiveness was internally assessed using three metrics: the area under the curve (AUC) of the ROC, calibration plot, and DCA. The statistical analyses were two-tailed and included 95% CIs. A *p*-value of less than 0.05 was deemed significant. All statistical analyses were conducted using SPSS version 22.0 (IBM, New York), R software,^1^ and Empower Stats software (www.empowerstats.com, X&Y Solutions, Inc., Boston, Massachusetts).

## Result

### Study participants

**Figure 1:**
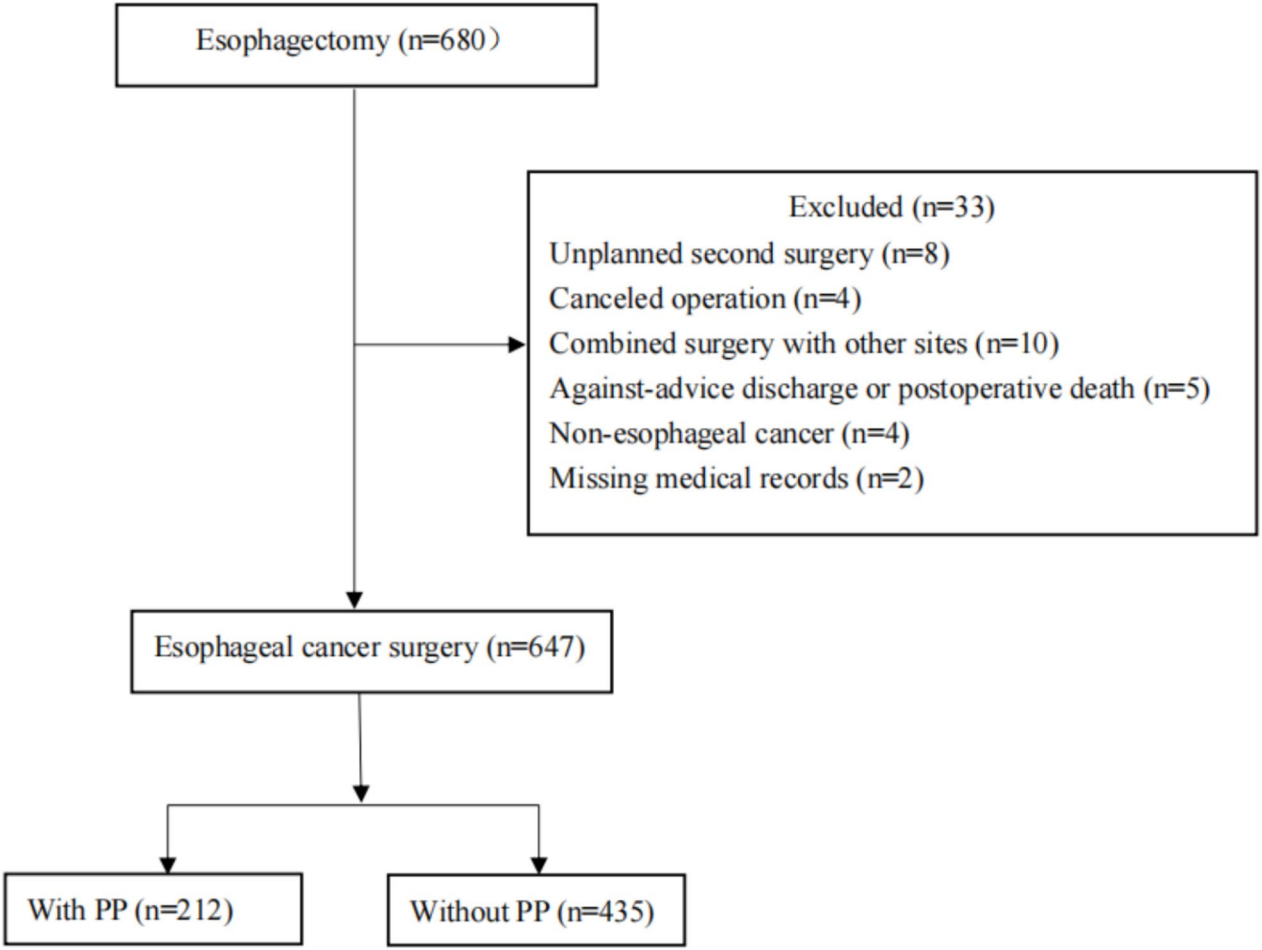
Flowchart of the process of patient enrollment. PP, Postoperative pneumonia.

Six hundred and forty-seven patients who had esophageal resections between January 1, 2010, and December 31, 2020, were ultimately included in this study. Thirty-three are excluded because of the following: unscheduled second surgery (*n* = 8); procedure cancelation (*n* = 4); multi-site combination surgery (*n* = 10); unplanned postoperative death or discharge (*n* = 5); postoperative pathological diagnosis of non-tumor (*n* = 4); and loss of medical records (*n* = 2) (Figure 1). Table 1 displayed the patient’s clinical and demographic details. With an average age of 61.06 ± 8.16 years, there were 647 patients (146 males and 353 females) and 212 (32.77%) of them developed PP (Table 1).

**Table 1:** The baseline characteristics of patients.

Variables	With PP (*n* = 212)	Without PP (*n* = 435)	*p*
Age (years), mean ±SD	61.86 ± 8.27	60.67 ± 8.09	0.082
Gender, *n* (%)			0.002
Female	32 (15.09%)	114 (26.21%)	
Male	180 (84.91%)	321 (73.79%)	
Smoking, *n* (%)	141 (65.51%)	201 (46.21%)	<0.001
Drinking, *n* (%)	77 (36.32%)	112 (25.75%)	0.006
Hypertension, *n* (%)	34 (16.04%)	68 (15.63%)	0.894
Diabetes, *n* (%)	11 (5.19%)	36 (8.28%)	0.156
Pulmonary diseases, *n* (%)	61 (28.77%)	89 (20.46%)	0.019
Neoadjuvant chemotherapy, *n* (%)	53 (25.00%)	89 (20.46%)	0.190
Hb (g/L), mean ±SD	130.92 ± 14.85	130.84 ± 15.25	0.949
ALB (g/L), mean ±SD	39.38 ± 4.38	39.76 ± 4.17	0.293
ALB (g/L), *n* (%)			0.233
<35	27 (12.74%)	42 (9.66%)	
≥35	185 (87.26%)	393 (90.34%)	
ASA status, *n* (%)			0.101
1	9 (4.25%)	11 (2.53%)	
2	180 (84.91%)	394 (90.57%)	
3	23 (10.84%)	30 (6.90%)	
Tumor location, *n* (%)			0.287
Upper	19 (8.96%)	25 (5.75%)	
Middle	153 (72.17%)	331 (76.09%)	
Lower	40 (18.87%)	79 (18.16%)	
Type of anesthesia, n (%)			
E-GA	154 (72.64%)	308 (70.80%)	
GA	58 (27.36%)	127 (29.20%)	
Type of ETT, *n* (%)			0.005
DLET	145 (68.40%)	248 (57.01%)	
SLET	67 (31.60%)	187 (42.99%)	
Continuous anesthesia, *n* (%)			0.016
TIVA	184 (86.79%)	403 (92.64%)	
CIIA	28 (13.21%)	32 (7.36%)	
Vasoactive drug use, *n* (%)	125 (58.96%)	216 (49.66%)	0.026
Surgery method, *n* (%)			0.118
MIE	89 (41.04%)	211 (48.51%)	
OE	123 (58.02%)	224 (51.49%)	
OT (min), mean ±SD	240.77 ± 57.02	238.34 ± 56.62	0.609
OT (min), *n* (%)			0.876
<240	119 (56.13%)	247 (56.78%)	
≥240	93 (43.87%)	188 (43.22%)	
PFV (mL), *n* (%)			0.830
≤2,000	65 (30.66%)	137 (31.49%)	
>2,000	147 (69.34%)	298 (68.51%)	
EBL (mL), *n* (%)			0.685
≤200	133 (62.74%)	280 (64.37%)	
>200	79 (37.26%)	155 (35.63%)	
PCA, *n* (%)			0.396
PCEA	155 (73.11%)	304 (69.89%)	
PCIA	57 (26.89%)	131 (30.11%)	

PP, postoperative pneumonia; Hb, Hemoglobin; ALB, albumin; ASA, American Society of Anesthesiologist; E-GA, combined epidural-general anesthesia; GA, general anesthesia; ETT: endotracheal tube; DLET, double lumen endotracheal tube; SLET, single lumen endotracheal tube; TIVA, total intravenous anesthesia; CIIA, combined intravenous and inhalation anesthesia; OE, open esophagectomy; MIE, minimally invasive esophagectomy; OT, operation time; PFV, perioperative fluid volume; EBL, estimated blood loss; PCA, patient controlled analgesia; PCIA, patient controlled intravenous analgesia; PCEA, patient controlled epidural analgesia.

### Screening of predictive factors

**Figure 2:**
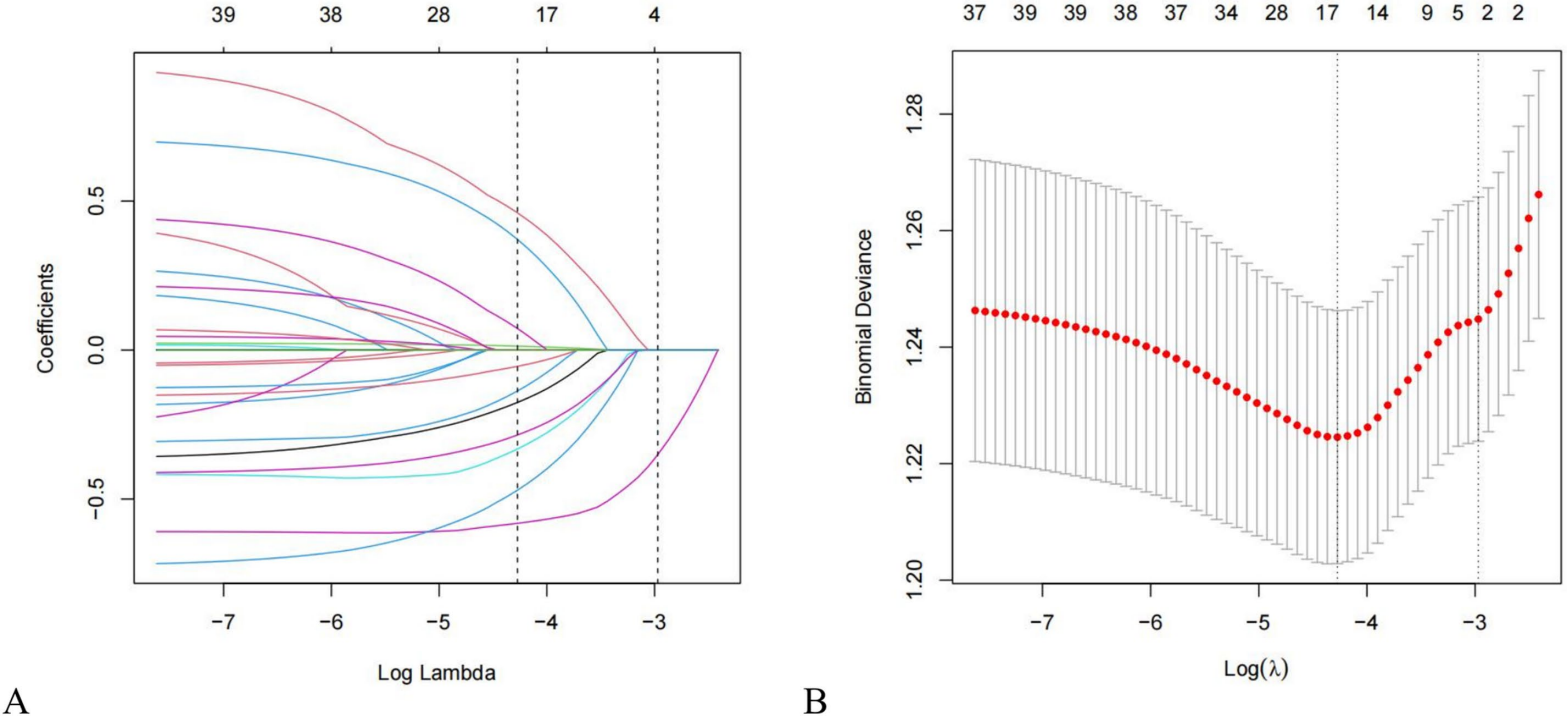
Features selection using the LASSO regression method. **(A)** LASSO coefficient profiles of 21 factors. Changes in clinical related factors and penalty parameters (*λ*); **(B)** The partial likelihood deviance (binomial deviance) curve was plotted versus Log (Lambda). Based on cross validation and minimum criteria, 21 factors with penalty parameter (λ) in the model were adjusted. LASSO, least absolute shrinkage and selection operator.

Twenty-one variables were involved to analysis and identify their association with PP. We utilized LASSO regression to identify key predictive factors for PP. Following the analyses, the factors independently related to PP were age (*p* = 0.029), smoking (*p* = 0.001), type of ETT (*p* = 0.001), continuous anesthesia (*p* = 0.014), and vasoactive drug usage (*p* = 0.003), as shown in Figures 2A,B and Table 2. Initially, five potential predictive factors were pinpointed via LASSO regression and are detailed in Table 2.

**Table 2:** Multivariate logistic regression analysis of the risk factors screened by LASSO regression.

Variables	adj. OR (95%CI)	*p*
Age	1.03 (1.00, 1.05)	0.029
Smoking	1.94 (1.29, 2.90)	0.001
Type of ETT		0.001
DLET	Reference	
SLET	0.52 (0.35,0.76)	
Continuous anesthesia		0.014
TIIA	Reference	
CIIA	2.03 (1.15, 3.59)	
Vasoactive drug use	1.59 (1.11, 2.29)	0.012

LASSO, least absolute shrinkage and selection operator; adj, adjusted; OR, odds ratio; CI, confidence interval; ETT, endotracheal tube; DLET, double lumen endotracheal tube; SLET, single lumen endotracheal tube; TIVA, total intravenous anesthesia; CIIA, combined intravenous and inhalation anesthesia.

### Nomogram for PP

**Figure 3:**
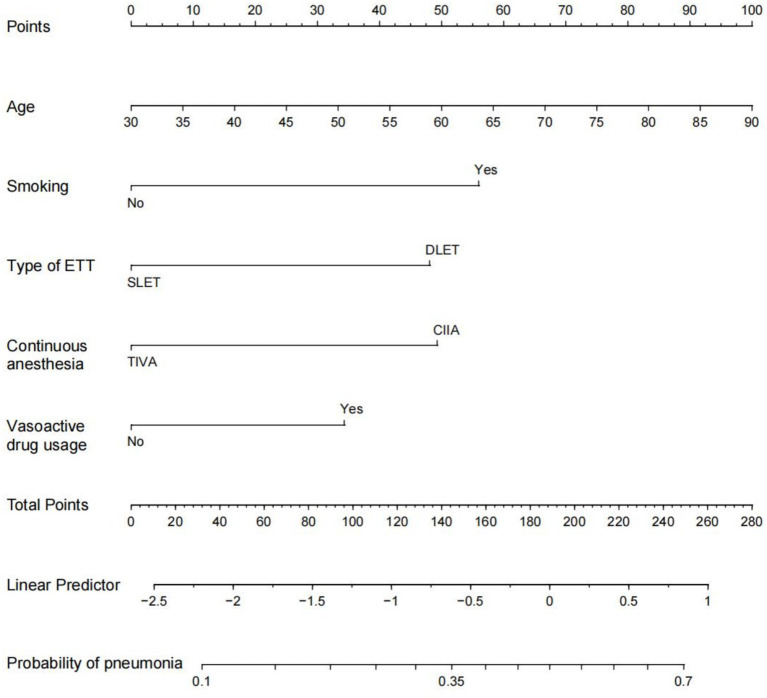
Nomogram prediction of postoperative pneumonia. The steps are: Determine the value of the variable on the corresponding axis, draw a vertical line to the total points axis to determine the points, add the points of each variable, and draw a line from the total point axis to determine the PP probabilities at the lower line of the nomogram. For example, a 60-year-old male with a history of smoking was treated with a double-lumen tracheal catheter and total intravenous anesthesia, and vasoactive drugs were used during the operation. According to the nomogram, the scores of each item were 50,56,48,0 and 33 in sequence. The total points are 187, and the corresponding incidence of postoperative pneumonia was 0.5 (50%). ETT: endotracheal tube. DLET, double lumen endotracheal tube; SLET, single lumen endotracheal tube; TIVA, total intravenous anesthesia; CIIA, combined intravenous and inhalation anesthesia.

To develop a prediction model for PP in esophageal cancer patients, we utilized predictive factors and created a visual tool known as a nomogram (Figure 3). Within this nomogram, each patient’s individual variables were assigned specific points on their respective axes. The cumulative sum of these points was then plotted on a total point axis, which correspondingly indicated the likelihood of experiencing PP.

### Model assessment and validation

**Figure 4:**
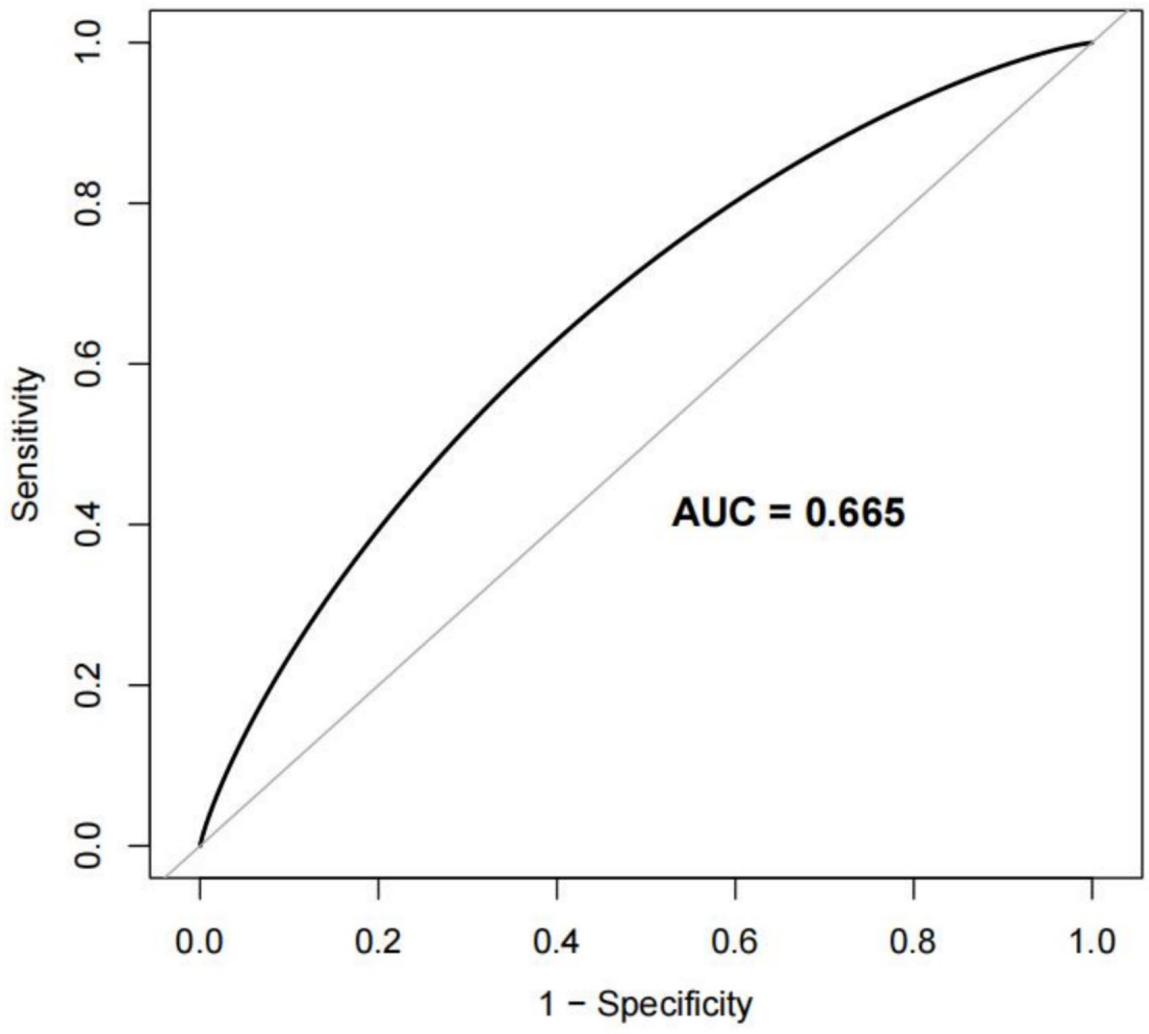
Receiver operating characteristic (ROC) curve for the prediction model Area under the curve (AUC). The AUC value ranges from 0.5 to 1. The closer it is to 1, the better the diagnostic performance and the higher the accuracy of the model.

**Figure 5:**
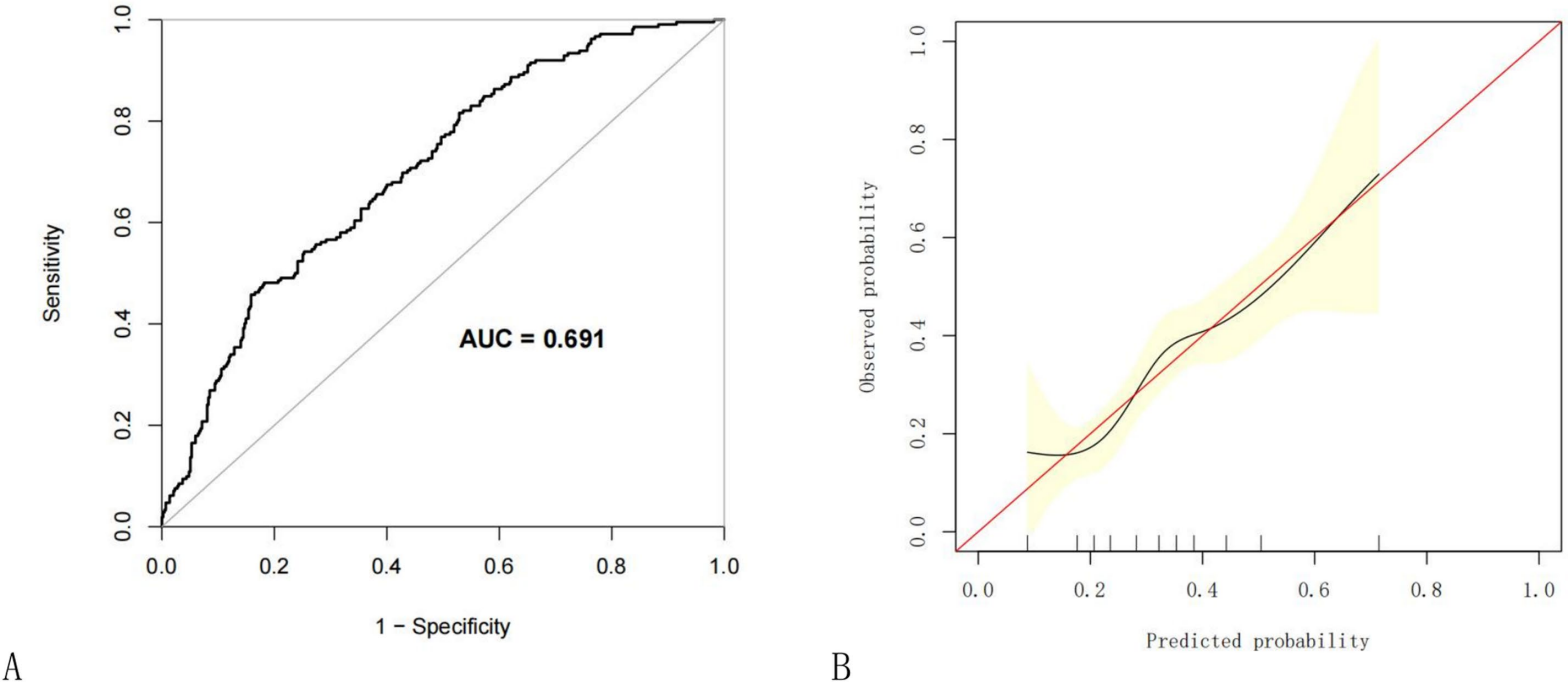
Internal validation of the nomogram using the bootstrap method with 500 resamples. **(A)** The ROC curve was measured by bootstrapping for 500 repetitions, and the AUC of the bootstrap stepwise model was showed; **(B)** Calibration curve for predicted probability of the pneumonia nomogram. The X axis is the predicted probability of the nomogram, and the Y axis is the observed probability. The red line shows the ideal calibration line and the black line is the curve fitting line, while the yellow area shows the 95% confidence interval of the prediction model. For a well-calibrated model, the black line should be arranged along the red line, while the farther away from the red line, the worse the calibration. AUC, Area under the receiver operating characteristic curve; ROC, Receiver operating characteristic.

**Figure 6:**
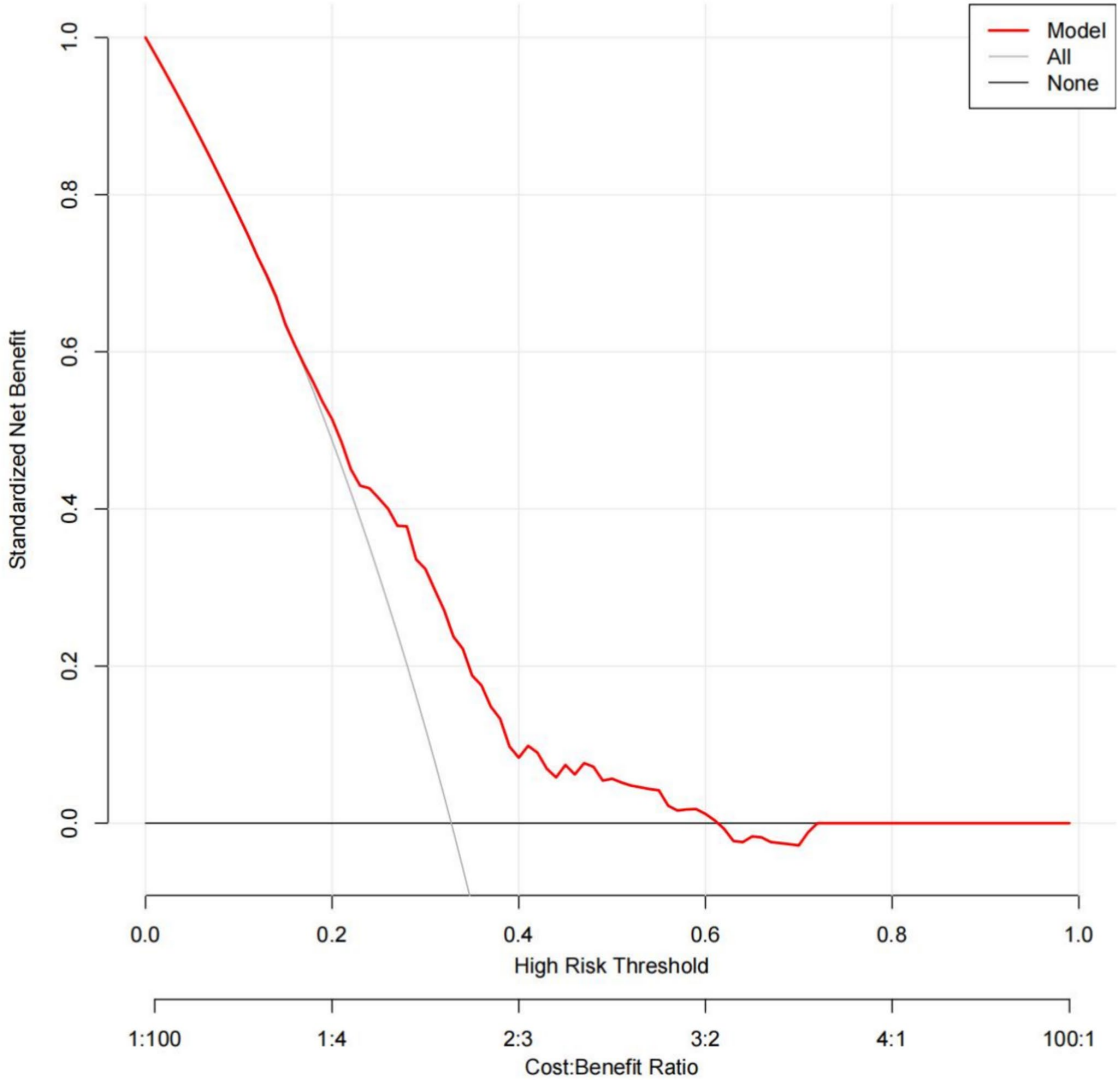
Decision curve analysis for the prediction model. Red solid line: Prediction model. Gray slash line: All patients received intervention. Solid horizontal line: None of the patients received intervention. The graph indicates the expected net benefit per patient relative to the nomogram prediction of pneumonia. When the threshold probability is from 0.17 to 0.61, the net benefit curve of the model is always higher than “All” and “None,” it indicates that the model has clinical value within a certain range of thresholds.

To evaluate the nomogram’s discrimination, ROC curve analysis was employed. An AUC (0–1) > 0.5 showed more accuracy; the closer the AUC is near 1, the better the forecast. With an area under the ROC curve (AUC) of 0.665 (95% CI: 0.620–0.704), this model demonstrated a diagnostic performance that was deemed to be quite satisfactory (Figure 4). In this study, the nomogram was validated using internal bootstrap validation. With a statistical power similar to the original stepwise model, the bootstrap stepwise model’s AUC was 0.691 (95%CI: 0.654–0.726) (Figure 5A). Through 500 iterations of bootstrapping, the ROC curve was assessed. The calibration curve was also used to assess the degree of similarity between the actual and expected risks. It was discovered that the estimated risk and the actual incidence of PP agreed well (Figure 5B). Overall, we were satisfied with the calibration and fit of our model to the ideal curve. When the threshold probability is from 0.17 to 0.61, the net benefit curve of the model is always higher than “All” and “None,” it indicates that the model has clinical value within a certain range of thresholds. Decision curve analysis, further demonstrated the predictive model’s good prospective clinical effect (Figure 6).

## Discussion

In this study, we assessed 21 relevant PP factors in 647 esophageal resection patients. Independent risk variables were age, smoking history, DLET, CIIA and vasoactive drugs usage. The perioperative surgical anesthesia factors were be predictors and were added into model for the first time, and a relatively comprehensive prediction model for postoperative pneumonia of esophageal cancer was established, which is presented in the form of a straightforward and user-friendly prediction nomogram. For the nomogram, five predictors were screened out by LASSO regression. This nomogram was internally evaluated using the bootstrap sampling technique and had high diagnostic performance (AUC = 0.691). Additionally, this prediction model fared better in the clinical setting (threshold probability is from 0.17 to 0.61) according to the decision curve analysis results.

Understanding the incidence of PP may be crucial for creating novel strategies to stop or lessen its occurrence. In the current study, 212 people in the current research had a PP diagnosis, which represents 32.77% of all esophageal resection patients. The incidence rates of postoperative pneumonia are somewhat high as compared to previous studies, yet these conclusions are unassailable since they are based on actual data. Publications indicate that the incidence of postoperative pneumonia following esophagectomy in esophageal carcinoma varies from 17.7 to 38% (17–21). Consequently, the incidence of postoperative pneumonia is high but yet within a reasonable range.

Our findings show that age, smoking history, DLET, CIIA and vasoactive drugs usage are significant and independent risk factors for PP in patients after esophagectomy in esophageal carcinoma. Our research shows that for every additional year of age in patients after esophageal cancer surgery, the risk of postoperative pneumonia increases by 3%. As individuals age, their physical functions tend to decline gradually. Specifically, the defense mechanisms of lung tissue in the elderly are weakened. Moreover, the immune system also deteriorates with age, and the function of alveolar macrophages, which play a crucial role in immune responses, is diminished (22). These factors collectively increase the risk of postoperative pneumonia in elderly individuals. Chronic smoking damages the airway mucosa’s ciliary structure, which reduces its capacity to clear mucus. Compared to nonsmokers, smokers have a higher risk of lung infections and airway blockages (23–26). According to a systematic study and meta-analysis, quitting smoking four to eight weeks before to surgery decreased the risk of pulmonary issues following surgery by 23 to 47% (27). In contrast to DLET anesthesia, SLET anesthesia significantly decreased the risk of postoperative pneumonia after esophageal surgery, according to our research. This conclusion was supported by a multicenter case–control study including 137 participants (28). When compared to DLET, SLET may reduce the incidence of postoperative pneumonia for the reasons listed below. First, SLET lowers intrapulmonary shunt, increases lung oxygenation, and reaches TLV. Second, DLET for OLV may result in bilateral inflammatory response, hypoxia-reoxygenation, and ischemia–reperfusion (29). Consequently, DLET has a higher risk of postoperative pneumonia than SLET. In practical practice, anesthesiologists select the right endotracheal tube type to satisfy surgeons’ needs, which lowers the risk of pulmonary problems following surgery. This is predicated on maintaining airway patency, sufficient breathing, and appropriate oxygenation. In this study, PP was more common in individuals with CIIA than in those with TIVA. However, a recent clinical study found no discernible difference in PP between the intravenous anesthetic propofol and volatile anesthetics desflurane or sevoflurane administered after lung surgery (30). Whether volatile anesthetics can lower PP in comparison to intravenous anesthetics is still up for debate (31, 32). Vasoactive drugs are frequently used as anesthetics during surgery for esophageal cancer. In contrast to what we discovered with esophagectomy, the use of vasoactive medications has been shown to decrease postoperative complications and length of hospital stay in abdominal surgery (33). One explanation could be because it is uncertain how much fluid infusion amount affects PP in cases with esophageal cancer (34). In this clinical trial, the vasoactive drug was used to keep blood pressure stable, but intraoperative fluid administration was the same for both groups. Fluid volume deficiencies in patients on vasoactive drugs may impact the pulmonary circulation and cause surgical pneumonia. Therefore, it is beneficial to reduce the incidence of PP by stopping smoking before to surgery, using the appropriate anesthetic medications during surgery, and learning when to use vasoactive drugs.

The nomogram was used in this investigation to measure the total likelihood of PP for every subject. Risk assessment, better patient-physician communication, and therapeutic decision-making can all benefit from this prediction model. In the current study, five independent factors were eliminated using LASSO regression, and the nomogram was then created to predict the chance of PP in EC patients. In terms of diagnostic performance, the nomogram did rather well (AUC = 0.660). This is the first predictive model in EC patients that can preliminarily assess the incidence of postoperative pneumonia based on the surgical anesthesia plan before the operation. A statistical method for determining the overall risk of PP in individuals who have had esophageal resection is the nomogram. The ARISCAT index is currently a commonly used quantitative table for postoperative pulmonary risk by evaluating seven objective indicators. Although it is specific, it may be troublesome for some busy clinicians. On the contrary, the nomogram is more convenient to apply, especially after we developed a simple web calculator.^2^ A significant early warning indication of PP for patients with esophageal cancer may be provided by this innovative nomogram. By applying the prediction model, if the probability of predicting postoperative pneumonia is between 0.17 and 0.61, it is beneficial to take intervention measures, such as optimizing the surgical anesthesia management plan, postoperative care, the use of preventive antibiotics and respiratory function exercises, etc.

This study offers several advantages. First, whereas most previous research looked at the prevalence of PP in patients with colonic or rectal cancer, very few studies were conducted among patients with EC. This study provided new evidence for PP in EC. Second, the predictive factors of the model in this study include many perioperative surgical anesthesia factors and can be used before the operation. Furthermore, decision curve analysis and internal bootstrap validation demonstrated that the nomogram was consistent and had good positive net advantages. There are some limitations in this study. First, because of the study’s retrospective nature, it could have a potential selection bias. A prospective research verification is needed in the future. Second, the sample size is relatively small, and a larger sample size is needed for demonstration, although the sample size of this study is enough. Third, due to the limitations of the model establishment method, it may lead to overfitting and instability. Meanwhile, the lack of external verification limits its universality. External validation can be incorporated into future research to promote the stability and universality of the model. Fourth, we left out a few unidentified possible factors that may have affected our findings. Possible confounding variables not captured in your dataset such as preoperative pulmonary rehabilitation, inhaler use, or severity of chronic lung disease.

## Conclusion

Age, smoking history, DLET, CIIA and vasoactive medication usage are independent risk variables of postoperative pneumonia after esophagectomy. Using the right anesthetics and ETT type during surgery, quitting smoking before the procedure, and knowing when to apply vasoactive medications are all ways to lower the risk of postoperative pneumonia. An intuitive nomogram model was developed in this study to predict PP in esophageal cancer patients. By predicting a crucial early warning indicator, the new nomogram may enable medical professionals to take the necessary precautions. External verification and larger-scale prospective studies are still needed in the future to verify this conclusion.

## Data Availability

The raw data supporting the conclusions of this article will be made available by the authors, without undue reservation.

## Footnotes

^1^ http://www.rproject.org

^2^ https://aoyawenlv.github.io/calculator/

## Glossary

EC: Esophageal carcinoma
PP: Postoperative pneumonia
OE: Open esophagectomy
MIE: Minimally invasive esophagectomy
GA: General anesthesia
E-GA: Combined epidural-general anesthesia
DLET: Double lumen endotracheal tube
SLET: Single lumen endotracheal tube
TIVA: Total intravenous anesthesia
CIIA: Combined intravenous and inhalation anesthesia
EPV: Events per variable
ALB: Albumin
Hb: Hemoglobin
OT: Operation time
ASA: American Society of Anesthesiologist Physical Status
ETT: Endotracheal tube
PFV: Perioperative fluid volume
EBL: Estimated blood loss
PCA: Patient controlled analgesia
PCIA: Patient controlled intravenous analgesia
PCEA: Patient controlled epidural analgesia
VIF: Variance inflation factor
LASSO: Least absolute shrinkage and selection operator
ROC: Receiver operating characteristic
DCA: Decision curve analysis
AUC: Area under the curve
CI: Confidence intervals.
